# Pathogenesis of Lassa Fever

**DOI:** 10.3390/v4102031

**Published:** 2012-10-09

**Authors:** Nadezhda E. Yun, David H. Walker

**Affiliations:** Department of Pathology, University of Texas Medical Branch, Galveston, Texas, USA; Email: neyun@utmb.edu (N.Y.); dwalker@utmb.edu (D.W.)

**Keywords:** arenavirus, Lassa virus, pathogenesis, cell-mediated immunity

## Abstract

Lassa virus, an Old World arenavirus (family Arenaviridae), is the etiological agent of Lassa fever, a severe human disease that is reported in more than 100,000 patients annually in the endemic regions of West Africa with mortality rates for hospitalized patients varying between 5-10%. Currently, there are no approved vaccines against Lassa fever for use in humans. Here, we review the published literature on the life cycle of Lassa virus with the specific focus put on Lassa fever pathogenesis in humans and relevant animal models. Advancing knowledge significantly improves our understanding of Lassa virus biology, as well as of the mechanisms that allow the virus to evade the host’s immune system. However, further investigations are required in order to design improved diagnostic tools, an effective vaccine, and therapeutic agents.

## 1. Phylogeny and geographic distribution

Lassa virus, the causative agent of Lassa fever, belongs to the family *Arenaviridae*. Arenaviruses are classified as segmented negative-sense RNA (nsRNA) viruses and are phylogenetically closely related to other segmented nsRNA viruses belonging to *Bunyaviridae* and *Orthomyxoviridae*.The three virus families share basic features of the intracellular replication cycle. Based on serological cross-reactivity [1], phylogenetic relations [2], and geographical distribution, all arenaviruses are further sub-divided into the Old World and New World virus complexes. The New World arenavirus complex comprises viruses that circulate in North America (i.e., Whitewater Arroyo (WWAV), Tamiami (TAMV), and Bear Canyon (BCNV) viruses) and South America (i.e., Tacaribe (TACV), Junin (JUNV), Machupo (MACV), Guanarito (GTOV), and Sabia (SABV) viruses). The Old World complex includes arenaviruses that circulate in Africa, Europe, and Asia (i.e., lymphocytic choreomeningitis (LCMV) and Lassa (LASV) viruses). The range of reservoir rodent species restricts the geographic occurrence of arenaviruses, with the exception of LCMV that is distributed worldwide owing to its association with *Mus domesticus *and *M. musculus, *which have migrated globally (Table 1). TACV is the only arenavirus that has been isolated from fruit-eating bats. The reservoir hosts of LASV are rodents of the genus *Mastomys *that are enzootic in sub-Saharan Africa [3]. In LASV endemic regions, up to 30% of *Mastomys *rodents can carry the virus [4]. There is strong phylogenetic evidence supporting the hypothesis that the diversity of arenaviruses resulted from a long-term co-evolutionary relationship with rodents of the family *Muridae* [2,5]. At least seven arenaviruses are known to cause severe hemorrhagic fever in humans, among which are LASV, JUNV, MACV, GTOV, and SABV that are endemic in West Africa, Argentina, Bolivia, Venezuela, and Brazil, respectively [6], and recently discovered Lujo (LUJV) and Chapare (CHAPV) viruses that originated in Zambia and Bolivia, respectively [7,8] . These viruses, except SABV, LUJV, and CHAPV are included in the NIAID’s Category A Priority Pathogens list, and all experimental work with these agents is only permitted in Biosafety Level 4 (BSL-4) facilities.

## 2. Virion structure, genome organization and expression

Arenaviruses have pleomorphic virions from 40 to more than 200 nm in diameter that consist of nucleocapsid surrounded by a lipid envelope [10]. On electron micrographs the interior of virions shows a characteristic granular appearance due to incorporation of host cell ribosomes in virus particles during assembly. This, yet unexplained, phenomenon was the basis for the family name ( arenosus = sandy). The genome of arenaviruses consists of two single-stranded RNA segments, small (S) and large (L). Both genomic segments have an ambisense gene organization and encode two genes in opposite orientation. The L RNA (~7 kb) encodes the viral RNA-dependent RNA polymerase (L) and the small RING finger zinc-binding protein (Z). The S RNA (~3.4 kb) encodes the glycoprotein precursor protein (GPC) and the nucleoprotein (NP). GPC is posttranslationally cleaved to yield two envelope glycoproteins GP1 and GP2 and the stable signal peptide (SSP).

**Table 1:** *Arenaviridae *family (adapted from reference [9]).

Virus	Acronym	Distribution	Reservoirs	Human Pathogen	Mortality Rate
**Old World arenaviruses**					
Ippy	IPPYV	Central African Republic	*Arvicanthis sp.*	not reported	
Lassa	LASV	West Africa	*Mastomys sp.*	Yes	High
Lymphocytic choriomeningitis	LCMV	Europe, Americas	*Mus musculus*	Yes	Low
Mobala	MOBV	Central African Republic	*Praomys sp.*	not reported	
Mopeia	MOPV	Mozambique, Zimbabwe	*Mastomys natalensis*	not reported	
**New World arenaviruses**					
Allpahuayo	ALLV	Peru	*Oecomys bicolor, Oecomys paricola*	not reported	
Amapari	AMAV	Brazil	*Oryzomys capito, Neacomys guianae*	not reported	
Bear Canyon	BCNV	USA	*Peromyscus californicus*	not reported	
Chapare	CHPV	Bolivia	unknown	Yes	unknown
Cupixi	CPXV	Brazil	*Oryzomys sp.*	not reported	
Flexal	FLEV	Brazil	*Oryzomys spp.*	Yes	non-fatal
Guanarito	GTOV	Venezuela	*Zygodontomys brevicauda*	Yes	High
Junin	JUNV	Argentina	*Calomys musculinus*	Yes	High
Latino	LATV	Bolivia	*Calomys callosus*	not reported	
Machupo	MACV	Bolivia	*Calomys callosus*	Yes	High
Oliveros	OLVV	Argentina	*Bolomys obscurus*	not reported	
Parana	PARV	Paraguay	*Oryzomys buccinatus*	not reported	
Pichinde	PICV	Colombia	*Oryzomys albigularis*	not reported	
Pirital	PIRV	Venezuela	*Sigmodon alstoni*	not reported	
Sabia	SABV	Brazil	unknown	Yes	unknown
Tacaribe	TACV	Trinidad	*Artibeus spp.*	Yes	non-fatal
Tamiami	TAMV	USA	*Sigmodon hispidus*	not reported	
Whitewater Arroyo	WWAV	USA	*Neotoma albigula*	Yes	Low
**Status remains pending**					
Lujo	LUJV	Zambia, South Africa	unknown	Yes	High
Luna	LUNV	Zambia	*Mastomys natalensis*	not reported	
Lunk	LNKV	Zambia	*Mus minutoides*	not reported	
Kodoko	KODV	Africa	*Mus minutoides*	not reported	
Dandenong	DANV	unknown	unknown	not reported	
Merino Walk	MWV	South Africa	*Myotomys unisulcatus*	not reported	

The enzymatic machinery for RNA synthesis in arenaviruses is contained within a single L polymerase protein. This 250-450 kDa protein utilizes viral RNA templates that consist of genomic RNA encapsidated by the viral nucleocapsid protein NP and comprises viral ribonucloprotein (RNP) [10]. L polymerase of arenaviruses contains the SDD motif characteristic of all RNA-dependent RNA polymerases (RdRp). Upon infection, once the virus RNP is delivered into the cytoplasm of the host cell, the L polymerase associated with the viral RNP initiates transcription from the genome promoter located at the 3’-end of each genomic RNA segment, L and S. The 5’ and 3’ terminal 19 nt viral promoter regions of both RNA segments required for the recognition and binding by the viral polymerase [11] exhibit a high degree of conservation among arenaviruses. The genome segments have highly complementary 5’- and 3’-ends (19 nt) that have been predicted to form panhandle structures [12]. The primary transcription results in the synthesis of mRNA of viral genes encoded in antigenomic orientation, NP and L polymerase, from the S and L segments, respectively. Transcription terminates at the distal side of the stem-loop (SL) structure within the intergenomic region (IGR). This SL structure has been proposed to stabilize the 3’-termini of the viral mRNAs [13,14]. Arenaviruses utilize a cap snatching strategy to acquire the cap structures of cellular mRNAs. Cap snatching is mediated by the endonuclease activity of the L polymerase that is co-factored by the cap binding activity of NP [15,16,17]. Therefore, arenaviruses produce capped non-polyadenylated mRNAs. Subsequently, the L polymerase adopts a replicase mode and moves across the IGR to generate a full-length complementary antigenomic RNA (agRNA). This agRNA serves as a template for the synthesis of mRNAs of viral genes encoded in genomic orientation, GPC and Z, from the S and L segments, respectively, and for the synthesis of full-length genomic RNA (gRNA) [10]. Both gRNA and agRNA of arenaviruses contain a nontemplate G residue at their 5’-ends [18]. The proposed “prime and realign” mechanism includes the synthesis of a pppG_P_C_OH_ dinucleotide primer from the CG nucleotides at positions +2 and +3 of the 3’-end genome promoter sequence, that is then realigned such that its 3’-terminal C_OH_ is opposite the genome 3’-terminal G residue, and the realigned pppG_P_C_OH_ then acts as a primer for a complementary RNA strand synthesis [19,20]. The matrix protein Z is not required for viral genome transcription and replication; however, Z exhibits a dose-dependent inhibitory effect on viral RNA synthesis [21,22,23]. This inhibitory effect of Z has been reported for New World [24], as well as for Old World [25] arenaviruses. 

In addition to the functions required to support virus replication, at least two viral proteins, NP and Z, have been proposed to modulate the host cell response to infection. NP is the most abundant viral protein both in infected cells and in virions, comprises the main structural component of the viral ribonucleoprotein (RNP) and plays an essential role in the synthesis of viral RNA [10]. Recent experimental data indicate that NP is involved in virus-induced inhibition of type I IFN signaling [26,27,28]. This activity has been mapped to the C-terminal domain of NP, which has a folding that mimics that of the DEDDH family of 3’-5’ exoribonucleases [29]. Functional analysis confirmed the exonuclease activity of LASV NP that has been proposed to be critical for its type I IFN counteracting function [15]. The small RING finger protein Z is the arenavirus counterpart of the matrix (M) protein of other negative sense RNA viruses [10]. Z protein of LCMV interacts with the promyelocytic leukemia (PML) protein and the eukaryotic translation initiation factor 4E (eIF4E) in infected cells and has been proposed to contribute to the noncytopathic nature of LCMV infection and repression of cap-dependent translation [30,31,32,33]. 

## 3. Epidemiology of Lassa fever

LASV was first isolated in 1969 from a missionary nurse who worked in a clinic in a small town, Lassa, in northeastern Nigeria [34]. The nurse presumably acquired infection from an obstetrical patient residing in Lassa. She died approximately one week after the onset of symptoms. Subsequently, two more nurses that attended the first patient contracted the disease, which was later named Lassa fever and caused the death of one of them. Infectious virus was isolated from all three cases [35].

Initially, several countries of West Africa were identified to be endemic for LASV, namely Sierra Leone [4,36], Guinea [37,38], Liberia [39,40,41], and Nigeria [42,43,44,45]. However, a serological survey among patients admitted with a history of fever and missionaries that had experienced a febrile illness showed that LASV was also present in Ivory Coast, Mali, and Central African Republic [46]. The notion that LASV was endemic in larger areas of West Africa was further supported by the results of investigation of an imported case of Lassa fever in Germany in 2000. During the incubation period, the index patient traveled through several countries, namely Ghana, Ivory Coast, and Burkina Faso, that were not considered to be endemic at that time [47]. Later, cases of Lassa fever have been reported from Burkina Faso, Ivory Coast, Ghana, Senegal, Gambia, and Mali [48,49].

According to estimations, LASV is responsible for 100,000-300,000 infections and approximately 5,000 deaths annually [50]. However, the high degree of seroprevalence of LASV-specific antibodies in the general population residing in the endemic regions, although highly variable depending on the geographical location (from 1.8% to 55%) [4,37,43,51], indicates that most infections are mild or possibly even asymptomatic and do not result in hospitalization. This is also supported by the findings indicating a high incidence of LASV-specific seroconversion, from 5% to 20% of the nonimmune population per year [4]. Nosocomial outbreaks are associated with higher mortality rates ranging from 36% to 65% [40,42,52]. However, serosurveillance studies in hospitals dealing with suspected Lassa fever cases showed that the hospital staff that routinely practiced basic hygiene measures had no higher risk of infection than the local population [53]. Infection with LASV presumably occurs through contact with body fluids or excreta, or inhalation of aerosols produced by infected animals. LASV is stable in aerosol [54], and animal-to-animal transmission via the airborne route has been demonstrated in the laboratory setting [55]. Hunting of peridomestic rodents and consumption of their meat is another important route of LASV transmission to humans residing in endemic areas of West Africa [38].

The multimammate mouse, *Mastomys natalensis*, was originally identified as the primary host species for LASV [56]. However, due to the poor understanding of the taxonomy of the genus, it is uncertain which species and particular subspecies serve as a reservoir for the virus [3]. The studies addressing the importance of *M. natalensis* for the circulation of LASV in nature demonstrated that newborn animals inoculated intraperitoneally develop persistent asymptomatic infection [57]. Significant infectious virus titers were detected in many organs, tissues, and fluids including lymph node, liver, spleen, lung, blood, and brain up to 74 days after inoculation. Moreover, LASV was isolated from the urine and throat swabs of infected animals. No significant histopathological alterations were observed in these animals. Interestingly, adult *M. natalensis* infected with LASV also developed a disseminated infection that lasted up to 30 days. Some animals cleared the virus from some organs, but there was persistence in other organs up to 103 days when the study was terminated. The only consistent histopathological finding observed in adult animals was a moderate chronic meningoencephalitis [57]. These data demonstrate that *M. natalensis* has an optimal pattern of infection and virus shedding for the maintenance of LASV in nature. 

## 4. Clinical description and pathogenesis of Lassa fever

The incubation period of Lassa fever ranges from 7 to 21 days [34,58]. The clinical disease begins as a flu-like illness characterized by fever, general weakness, and malaise, which may be accompanied by cough, sore throat, and severe headache. Gastrointestinal manifestations such as nausea, vomiting, and diarrhea are also common (Table 2). The differential diagnosis of Lassa fever based on the presenting symptoms can be problematic due to the many other acute undifferentiated febrile illnesses circulating in West Africa [58,59]. Although, hemorrhagic manifestations are not an important feature of Lassa fever, perturbation of vascular function is likely to be central to Lassa fever-associated pathobiology, since the signs of increased vascular permeability, such as facial edema and pleural and pericardial effusions, indicate a poor prognosis for the disease outcome. Recovery from Lassa fever generally begins within 8 to 10 days of disease onset. In severe cases, the condition of the patient deteriorates rapidly between the 6th and 10th day of illness with severe pulmonary edema, acute respiratory distress, clinical signs of encephalopathy, sometimes with coma and seizures, and terminal shock. Bleeding from mucosal surfaces is often observed; however, it is usually not of a magnitude to produce shock by itself [58]. Sensorineural deafness is commonly observed in patients in the late stages of disease or in early convalescence in survivors [60]. 

The level of viremia is highly predictive of the disease outcome. In a study involving 137 patients with Lassa fever, patients that presented with viremia < 10^3^ median tissue culture infectious dose (TCID_50_)/ml on the day of hospitalization had 3.7 times greater chance of survival than those admitted with higher levels of viremia. Similarly, the probability of fatal outcome in patients with serum titers > 10^3^ TCID_50_/ml and serum levels of aspartate aminotransferase (AST) ≥ 150 international units (IU)/L was 21 times higher than that in patients not meeting either of these criteria. Virtually all patients with fatal Lassa fever whose sera were tested were viremic at the time of death with terminal viremia ranging from 10^3^ to 10^8^ TCID_50_/ml [61]. Detailed studies have shown that viremia peaks between 4 and 9 days after the onset of symptomatic disease and is followed by pronounced clinical manifestations. Patients recovering from Lassa fever clear virus from blood circulation about 3 weeks after the beginning of illness [40,61,62,63]. 

The current knowledge of Lassa fever pathogenesis does not include the chain of events that take place during disease development and lead to death of severely ill patients. Apparently, failure to develop the cellular immune response that would control dissemination of LASV, which is indicated by high serum virus titers, combined with disseminated replication in tissues and absence of neutralizing antibodies, leads to the development of fatal Lassa fever [64]. However, considering the high mortality and truly dramatic course of the disease, the pathological findings do not provide the basis that would explain the mechanism of disease progression and the cause of death from Lassa fever.

**Table 2:** Onset and duration of the principal clinical manifestations of Lassa fever (adapted from reference [58]).

Clinical signs	Day of illness		Duration,
and symptoms	Start day	End day	days
Fever	1	11	10
Weakness	3	14	11
Cough	3	14	11
Chest pain	4	13	9
Back pain	4	12	8
Joint pain	4	12	8
Sore throat	4	11	7
Dysuria	4	10	6
Headache	4	11	7
Abdominal pain	5	8	3
Vomiting	5	9	4
Diarrhea	5	9	4
Pharyngitis	7	12	5
Conjunctivitis	7	12	5
Bleeding	7	11	4
Abdominal tenderness	9	14	5
Rales	9	14	5
Facial edema	9	16	7

Physical examination of patients after the onset of fever often reveals purulent pharyngitis, bilateral conjunctival hemorrhages, facial edema, and generalized abdominal tenderness. Macroscopic pathological changes can include pleural effusions, pulmonary edema, ascites, and hemorrhagic manifestations in the gastrointestinal mucosa [34,65]. Microscopic findings include hepatocellular necrosis and apoptosis, splenic necrosis, adrenocortical necrosis, mild mononuclear interstitial myocarditis without myocardial fiber necrosis, alveolar edema with capillary congestion and mild interstitial pneumonitis, lymph nodal sinus histiocytosis with mitoses, gastrointestinal mucosal petechiae, renal tubular injury, and interstitial nephritis [34,59,66]. A comprehensive postmortem histopathological examination of 21 virologically confirmed community-acquired cases of Lassa fever in Sierra Leone revealed [59] variable levels of hepatic necrosis involving from 1 to 40% of hepatocytes. The necrotic hepatocytes were randomly distributed often forming foci of contiguous cells. Mononuclear phagocytes were observed either contacting or phagocytosing necrotic hepatocytes. Interestingly, this phagocytic reaction, although highly variable from case to case and even from one necrotic focus to another in the same case, demonstrated a tendency towards homogeneity of the level of involvement within a particular patient. The predominant distribution of splenic necrosis was observed in the marginal zone of the periarteriolar lymphocytic sheath. Close examination of thin tissue sections revealed the presence of fibrin in addition to the debris of necrotic cells. Splenic venous subendothelium appeared to be infiltrated by lymphocytes and other mononuclear cells. Microscopic examination of adrenal glands showed prominent spherical, hyaline, acidophilic cytoplasmic inclusions in cells near the junction of zona reticularis and medulla. In most cases these cells appeared to be adrenocortical cells of the zona reticularis; however, some cells were of adrenal medulla origin. Additionally, multifocal adrenocortical cellular necrosis was detected that was most prominent in the zona fasciculata and was often associated with focal inflammatory reaction. However, in all examined cases adrenal necrosis was mild and ≥ 90% of the cells of adrenal cortex appeared viable [59].

The major and most common lesions of Lassa fever in humans occur in the liver [34,59,66,67]. There are four principal features of LASV hepatitis can be derived: 1) focal cytoplasmic degeneration of hepatocytes suggestive of phagocytosed apoptotic fragments; 2) randomly distributed multifocal hepatocellular necrosis; 3) monocytic reaction to necrotic hepatocytes; 4) hepatocellular mitoses. These morphologic effects do not uniformly occur in all cases, but in some instances can be found simultaneously [59,66]. Based on the degree of hepatic damage, three general nosopoetic phases have been proposed to divide patients with fatal Lassa fever into categories with respect to pathogenic events in fatal LASV hepatitis [66]. The first phase, active hepatocellular injury, is defined by the presence of focal cytoplasmic degeneration with <20% of hepatocytes undergoing necrosis. This phase may represent the late stage of viremic spread and early cellular injury, which is, most likely, caused by direct viral action rather than mediated by a cellular immune response, since lymphocytic infiltration is not detected. The second phase, the peak of Lassa hepatitis, is characterized by 20-50% necrosis of hepatocytes, widespread focal cytoplasmic degeneration and limited phagocytic infiltration. This is suggested not only progressive hepatocellular damage, but, also, early liver recovery through phagocytic removal of necrotic hepatocytes and regeneration of new cells. The third phase, hepatic recovery, is defined by <10% of hepatocellular necrosis, absence of focal cytoplasmic degeneration and clear evidence of mitoses, which indicate liver regeneration [66]. Interestingly, no correlation has been observed between the degree of hepatic necrosis and chemical indicators of liver damage, such as elevated levels of AST, alanine transaminase (ALT), and lactate dehydrogenase (LDH) in serum [59,66]. Overall it is apparent that the level of liver damage can vary dramatically in patients that die from Lassa fever. Therefore, it can be concluded that liver disease is a necessary, but not sufficient, condition in the chain of pathological events that lead to fatal outcome.

Lassa fever is not considered to be associated with coagulation dysfunction, e.g., neither decrease in the coagulation factors nor disseminated intravascular coagulation (DIC) has been observed in infected patients. However, moderate thrombocytopenia with significantly impaired functionality of thrombocytes is detected in patients with severe Lassa fever [68].

One possible mechanism involved in Lassa fever pathogenesis could be infection-triggered induction of uncontrolled cytokine expression similar to what is seen in sepsis. This hypothesis is supported by experimental data obtained from a case of fatal Lassa fever imported into Germany in 2000 [69]. In this patient, who died from multi-organ failure and hemorrhagic shock, the proinflammatory cytokines, interferon γ (IFN- γ) and tumor necrosis factor α (TNF- α), rose to extremely high levels shortly before death. However, in another study no elevation of both cytokine levels was observed in the examined fatal cases of Lassa fever, which suggests that the levels of IFN- γ and TNF- α are either elevated only in a fraction of patients or during a very short period that would require frequent sampling for detection [70]. 

Another possibility is that virus-induced immunosuppression may be involved in the pathogenesis of severe Lassa fever [71]. Thus, infection with LASV fails to activate monocyte-derived dendritic cells (DC) and macrophages (MP) of human origin. Infected DC fail to secrete proinflammatory cytokines, do not upregulate costimulatory molecules, such as CD40, CD80, and CD86, and poorly induce proliferation of T cells [72,73]. Importantly, human DC infected with the naturally nonpathogenic Mopeia virus, a closely related arenavirus that shares 75% amino acid similarity with LASV and was isolated from the same rodent reservoir [2], induces stronger CD4 and CD8 T-cell responses than those infected with LASV [74]. Downregulation of immune responses caused by LASV infection demonstrated in vitro is also in agreement with the results of clinical observations showing that fatal outcome of Lassa fever correlates with low levels or absence of interleukin (IL) 8 and IFN inducible protein 10 (IP-10) in circulation [70].

## 5. Immune responses to LASV infection

Patients infected with LASV produce IgM and IgG antibody isotypes [61]. However, since both immunoglobulin classes are detected in viremic patients, most likely the antibodies that are produced early in infection are not neutralizing. This is in agreement with reports showing that neutralizing antibodies appear months after acute infection is resolved, and the titers are often low [75]. Interestingly, the neutralizing antibody titers continue to rise even several months after convalescence has been established, which may indicate constant stimulation of B cells due to low levels of virus persistence. Antibodies in seroconverted individuals are specific to GPC, NP, and, likely, Z protein [76,77,78,79]. Experiments aimed at the identification of B-cell antigenic epitopes elucidated four sites on NP, two sites on GP1, and six sites on GP2 [80,81,82]. Some antibodies are highly LASV strain-specific, while other react with a broad range of arenaviruses including African and South American members of the family [80,81].

Since neutralizing antibodies appear in survivors long after virus has been cleared, it has been considered that resolution of LASV infection is mediated by cellular immunity. This hypothesis is supported by experimental data showing that cynomolgus monkeys that survive LASV infection have high concentrations of activated T lymphocytes and efficiently control viral replication. In contrast, the animals that succumb to infection display delayed, low-level T-cell activation, uncontrolled viral replication, and develop fatal disease [83]. Seropositive individuals residing in endemic areas have very strong memory CD4+ T-cell responses, and the antigenic epitopes are mapped to NP and a 13 amino acid region in the N-terminal part of GP2 that is highly conserved between Old and New World arenaviruses [84,85]. Importantly, a recent study showed that transgenic mice that express human major histocompatibility complex class I (MHC-I) molecules fail to control virus replication and are susceptible to Lassa virus, which is in contrast to normal laboratory mice that rapidly clear virus. Intriguingly, CD8 and CD4 T cell depletion in these mice prevents disease despite high levels of viremia [64]. These data indicate that T cells are essential for rapid resolution of LASV infection; however, if the host fails to control virus replication due to inadequate activation of the immune system, T lymphocytes may play a key role in Lassa fever pathogenesis.

## 6. Animal models of Lassa fever

Mice and guinea pigs have been evaluated as models of LASV infection [55]. However, normal adult mice are highly resistant to peripheral routes of inoculation. Mice expressing humanized MHC-I are LASV-susceptible and develop a severe illness [64]. Genotype of a particular mouse strain has significant influence on the development of pathologic manifestations in infected mice. Thus, newborn mice develop an asymptomatic infection upon inoculation with LASV with high virus titers in the brain, lung, and muscle, while intracranial inoculation of adult mice results in a fatal convulsive disorder resembling classical murine LCM immunopathology. However, other investigators failed to reproduce these findings [55,86,87]. Pathogenicity of LASV for guinea pigs largely depends on the host strain and the virus used for inoculation. For instance, Josiah strain of LASV causes a uniformly lethal disease in inbred strain 13 guinea pigs; however, the lethality varies from approximately 30% to more than 60% in outbred Hartley guinea pigs. Inbred animals have higher viral titers in all target tissues than outbred guinea pigs [57,88]. LASV-infected guinea pigs destined to die develop respiratory insufficiency with pulmonary edema, alveolar hyaline membranes, myocarditis, and focal calcification of myocardial fibers and hepatocytes. Terminally ill animals are viremic and contain virus in nearly every organ tested. There are two major differences between Lassa fever pathogenesis in humans and guinea pigs. In humans LASV is particularly hepatotropic, and patients with severe Lassa fever develop hepatocellular necrosis; however, in guinea pigs only foci of calcified hepatocytes are observed. On the other hand, LASV is myocardiotropic and myocardiopathic in guinea pigs, findings that have not been reported in humans [57]. Also, several isolates of LASV from clinical human cases are benign in guinea pigs, and some are lethal when tested in cynomolgus monkeys [89]. Therefore, in order to reliably model the pathogenesis of Lassa fever in humans, studies in non-human primates are required.

Several non-human primate species have been evaluated as potential models for Lassa fever including squirrel monkeys, capuchin monkeys, marmosets, hamadryas baboons, African green monkeys, cynomolgus monkeys, and rhesus monkeys [55,57,90,91,92,93,94,95]. Capuchin and squirrel monkeys seroconverted upon infection with LASV; however, the animals of both species uniformly survived low and high dose inoculations [55,57]. Virus was detected in virtually all organs of infected squirrel monkeys, but the liver, lymph nodes, and kidneys were the key early targets. Later in infection, the spleen, heart, and brain also showed pathological alterations. Viremia persisted in all animals for up to 28 days after infection. Histopathologic changes were mild and included germinal center necrosis in the spleen and lymph nodes, myocarditis, acute arteritis, renal tubular necrosis and regeneration, chronic inflammation of the choroid plexus, ependyma, and meninges, and cerebral perivascular cuffing. Hepatocytic regeneration suggesting recovery from disease-mediated liver damage was also observed [91]. Hamadryas baboons infected with LASV develop a disease that clinically resembled a severe form of human Lassa fever. The animals developed fever, characteristic pathologic and hemorrhagic manifestations, and high levels of viremia [93]. Interestingly, African green monkeys and rhesus macaques challenged with low doses (10-15 pfu) of LASV uniformly succumbed to the infection, whereas a high challenge dose (10^6^ pfu) was only partially lethal in these animals [55]. The gross pathological changes in LASV-infected rhesus macaques were petechiae and, in some animals, mild-to-moderate pleural effusions. Viremia appeared between days 5 and 10 in all animals and increased progressively until the animals died or met the criteria for euthanasia. Virus was detected in all organs tested, namely adrenal glands, spleen, liver, duodenum, jejunum, colon, bone marrow, lymph nodes, thymus, heart, lungs, pleural fluid, kidneys, skeletal muscle, pancreas, salivary glands, ovaries, bladder, cerebrum, cerebellum, brain stem, cerebrospinal fluid, and aqueous humor of the eye. The largest quantities of virus were generally detected in adrenal glands, spleen, liver, bone marrow, and intestines. The microscopic lesions included mononuclear pulmonary arteritis with intraluminal mononuclear cell aggregates and mononuclear cell infiltration of the subendothelium and the pulmonary arterial wall accompanied by endothelial hypertrophy and hyperplasia, hepatocellular necrosis, interstitial pneumonia, adrenal gland necrosis, encephalitis and uveitis. Therefore, infected rhesus monkeys exhibited pathologic lesions similar to human Lassa fever, such as the amounts and organ distribution of virus, necrosis of hepatocytes, adrenal cortical cells, and splenic marginal zone of the periaortic lymphocytic sheath, and interstitial nephritis. However, meningoencephalomeningitis, pulmonary vasculitis, systemic arteritis, and skeletal myositis were significantly more prominent in the monkey model than in human Lassa fever [92,96]. The clinical manifestations of LASV-infected cynomolgus macaques included fever, weight loss, depression, and acute respiratory syndrome. Other clinical features included thrombocytopenia, lymphopenia, lymphadenopathy, splenomegaly, infiltration of mononuclear cells, and pathologic alterations in the liver, lungs, and endothelium, which essentially mirrored observations from human cases [83]. An additional feature of the disease observed in cynomolgus monkeys was multifocal to severe central nervous system lesions at terminal stages. Dysregulation of the host immune response characterized by increased circulating levels of proinflammatory cytokines/chemokines including IL-1β, IL-6, MCP-1, and eotaxin were detected in the infected animals. Histopathologic evaluation of tissues revealed a sequence of events in LASV infection in cynomolgus macaques, which is initiated with dendritic cells in the lymphoid tissues, then progresses to infection of Kupffer cells in liver and parenchymal cells in liver and adrenal glands, and endothelial cells in various organs including the central nervous system [95]. Experimental infection of common marmosets results in systemic disease from clinical and pathologic standpoints highly similar to disease observed in fatal cases of Lassa fever in humans. Among the main clinical features are fever, weight loss, high viremia and viral RNA loads in tissues, elevated levels of liver enzymes, and severe morbidity between days 15 and 20. Histopathologic changes include multifocal hepatic necrosis associated with mild inflammation and hepatocyte proliferation, adrenal necrosis, lymphoid depletion, and interstitial nephritis. The necrotic hepatocellular foci observed in LASV-infected marmosets contained predominantly macrophages with the near absence of CD20-, CD8-, or CD3-positive lymphocytes, markedly decreased expression of MHC-II molecules, and hepatocyte proliferation. The levels of MHC-II expression were also significantly reduced in lymph nodes. Overall numbers of CD20- and CD3-positive lymphocytes in the spleen of infected animals were reduced, and the destruction of lymphoid follicular architecture was evident [94]. These findings strongly suggest that the replication of LASV in target tissues may cause pathologic changes that directly impair the adaptive immune response to LASV infection. 
